# Physical Activity and Metabolic Alterations in Children and Adolescents Across Different Weight Groups: A Systematic Review

**DOI:** 10.1002/ejsc.70206

**Published:** 2026-06-20

**Authors:** Marja H. Leppänen, Jukka E. Hintikka, Kathleen Wijnant, Eero A. Haapala, Timo A. Lakka, Lynn Vanhaecke, Heli Viljakainen

**Affiliations:** ^1^ Institute of Biomedicine School of Medicine University of Eastern Finland Kuopio Finland; ^2^ Folkhälsan Research Center Helsinki Finland; ^3^ Faculty of Medicine University of Helsinki Helsinki Finland; ^4^ Faculty of Sport and Health Sciences University of Jyväskylä Jyväskylä Finland; ^5^ Laboratory of Integrative Metabolomics (LIMET), Faculty of Veterinary Medicine Ghent University Ghent Belgium; ^6^ Nutrition Unit Faculty of Medicine and Health Sciences Ghent University Ghent Belgium; ^7^ Active Life Lab, Preventive Health Research Unit South‐Eastern Finland University of Applied Sciences Mikkeli Finland; ^8^ Department of Clinical Physiology and Nuclear Medicine Kuopio University Hospital University of Eastern Finland Kuopio Finland; ^9^ Kuopio Research Institute of Exercise Medicine Kuopio Finland; ^10^ Institute for Global Food Security School of Biological Sciences Queen's University Belfast Northern Ireland UK

**Keywords:** adiposity, exercise, metabolomics, pediatrics

## Abstract

Inadequate physical activity (PA) and increased sedentary time are key drivers of cardiometabolic disorders related to being overweight, and metabolomics offers a promising novel approach to study their associations. The aim of this systematic review was to assess the evidence on metabolites associated with PA and/or sedentary time among children and adolescents in different weight groups, integrating both intervention and observational studies to provide a comprehensive and broad synthesis of existing evidence. Following the Preferred Reporting Items for Systematic Reviews and Meta‐Analysis (PRISMA) guidelines, three databases (PubMed, Web of Science, and Scopus) were systematically searched for studies published from inception to December 2023 conducted in children and adolescents aged ≤ 18 years including metabolomics analyses focusing on PA, sedentary time, or cardiorespiratory fitness. Fifteen studies were included, and half of the studies were conducted in overweight individuals. Notable PA‐induced or PA‐associated alterations were seen in lipid, branched‐chain amino acid and ammonia metabolism, and the citric acid and glucose‐alanine cycles. The directions of the alterations seemed consistent across children and adolescents with normal body weight and overweight, but not in trained peers. Several metabolites and metabolite groups were identified as markers of higher PA and better cardiorespiratory fitness, reflecting favorable metabolic states in children and adolescents. However, there is still a great need for more in‐depth metabolomics studies using state‐of‐the‐art techniques in the fields of pediatric exercise science and public health. Training status, exercise modalities, and pubertal development are important covariates to consider in future studies.

## Highlights

- Based on the 15 studies retained, physical activity‐induced or physical activity‐associated alterations were involved in lipid, branched‐chain amino acid and ammonia metabolism as well as in the citric acid and glucose‐alanine cycles.
- The directions of the alterations seemed consistent across children and adolescents with normal body weight and living with overweight, but not in trained peers.
- Training status, exercise modalities, and pubertal development are important covariates to consider in studies investigating effects of physical activity on metabolism.

## 1 Introduction

The prevalence of overweight and obesity has multiplied over the last few decades, with more than 300 million children and adolescents now affected, making it a serious public health problem (NCD Risk Factor Collaboration (NCD‐RisC) 2017; World Health Organization 2021). Overweight and obesity are common risk factors for insulin resistance and type 2 diabetes from childhood onwards (Sahoo et al. 2015). If the development of excess adiposity is not prevented at an early age, an increased risk of cardiometabolic diseases follows into adulthood (Juonala et al. 2011).

Insufficient physical activity (PA) and increased sedentary time are two common drivers of cardiometabolic disorders associated with being overweight. It is generally accepted that increasing PA through play or sports activities is beneficial for the overall health of children and adolescents (Dimitri et al. 2020). Furthermore, improved cardiorespiratory fitness (CRF) has been noticed to lower cardiometabolic risk in children, especially in those living with overweight (Nyström et al. 2017; Haapala, Tompuri, et al. 2022). Sedentary time, conversely, is linked to the development of excessive weight gain, and likely, also to the development of adipose tissue dysfunction (Frodermann et al. 2019; Lee et al. 2019). Current PA guidelines recommend an average of 60 min of moderate‐to‐vigorous PA (MVPA) per day for 5–17‐year‐olds (WHO guidelines on physical activity, 2020), however, only a minority reaches these recommendations (Roman‐Viñas et al. 2016). Traditional cardiometabolic risk factors, such as central obesity, insulin resistance, hyperglycemia, dyslipidemia, and hypertension, can be poor biomarkers for the adverse effects of overweight mainly due to a lack of consensus on thresholds for children and adolescents. Therefore, novel biomarkers and omics studies are needed to fill these knowledge gaps (Herder et al. 2014). The physiological effects of PA on energy expenditure are well‐characterized, generally supporting a healthier body composition (Westerterp 2018). Yet, the mechanisms involved are not yet fully understood in individuals living with overweight, which can be further elucidated by global metabolomic fingerprinting (Butte et al. 2015; Bertram et al. 2009).

Metabolomics, the comprehensive analysis of low molecular weight compounds in a sample matrix, seeks to understand complex molecular interactions in biological systems (Fiehn 2002) and the effects of external stimuli, such as PA, often in a hypothesis‐free manner (Bertram et al. 2009). This facilitates the early detection of diseases and their risk factors and allows monitoring the effects of various therapies and interventions for health outcomes, such as overweight (Dunn et al. 2011; Jacob et al. 2019). Therefore, metabolomics offers an advantageous approach for capturing dynamic metabolic responses at an early age. Blood and urine are the most commonly used biofluids for metabolomics analyses (Bertram et al. 2009; Khoramipour et al. 2022), although the use of saliva as an easy‐accessible, non‐invasive option is increasing (Bosch 2014). Both options offer a child friendly alternative to venous blood draws and can be collected without extensive training. The most used analytical methods are liquid chromatography coupled to mass spectrometry (LC‐MS), gas chromatography coupled to mass spectrometry (GC‐MS), and nuclear magnetic resonance spectroscopy (NMR). Well‐designed studies combining exercise and metabolomics can provide a comprehensive picture into the relationship between physiology, lifestyles, and environment (Khoramipour et al. 2022). A recent review identified several consistent metabolic alterations related to childhood obesity, indicating that studying the metabolome at an early age may assist the early prediction of the development of overweight‐related disorders (De Spiegeleer et al. 2021). Yet, the mechanisms beyond PA in childhood overweight and obesity remain unclear.

To the best of our knowledge, there are no previous systematic reviews on the effects of PA or sedentary time on the metabolome in children and adolescents, especially with a focus on body weight. Since childhood overweight increases the risk of adverse cardiometabolic consequences in adulthood (Juonala et al. 2011), there is a need for deeper knowledge on PA‐related metabolic alterations in early age. In addition, it is of great interest whether PA can protect children and adolescents living with overweight from developing comorbidities of overweight. The aim of this study was to systematically review the literature on metabolites associated with PA and/or sedentary time in children and adolescents of different weight groups.

## 2 Materials and Methods

### 2.1 Data Sources and Search Strategy

This systematic review was conducted according to the Preferred Reporting Items for Systematic Reviews and Meta‐Analysis (PRISMA) 2020 statement (Page et al. 2021). The protocol for the review was registered in the International Prospective Register of Systematic Reviews (PROSPERO, id 366821). Literature on the subject published from inception to December 31^st^, 2023, was searched in three different databases: PubMed, Web of Science and Scopus. The search was made for original articles on children (aged < 10 years) and adolescents (aged 10–18 years) (World Health Organization 2023), where the topic included PA or corollary terms, metabolomic analyses, and weight or body composition. Search terms and strategies were appropriately modified for each database (Supporting Information S1: Table S1). Duplicate articles were removed manually by browsing the article metadata.

### 2.2 Study Selection and Quality Assessment

Screening and selection of articles were based on pre‐defined inclusion and exclusion criteria (Table 1) and were performed in duplicate by two authors (MHL and JEH). In case of discrepancies, the disagreements were resolved by team discussion. In addition, reference tracking of screened studies was used as a secondary source for articles. First, unique titles and abstracts were screened for eligibility (inter‐reviewer agreement = 98%) and then the resulting full texts were screened (inter‐reviewer agreement = 91%). Quality and risk of bias were assessed using the critical appraisal tools by the JBI (Aromataris 2022), specifically the checklists for analytical cross‐sectional studies, quasi‐experimental studies, and randomized controlled trials. Articles were scored based on the number of “yes” answers in the checklist divided by the number of items, not counting the “not applicable” items (Supporting Information S1: Table S2–S4).

**TABLE 1:** Inclusion and exclusion criteria for the studies screened for the systematic review.

	Study attributes
Inclusion criteria	
Study design	Population‐based observational studies and intervention studies
Population	Children and adolescents aged 18 years or less
Exposure	For intervention studies, studies focusing exclusively on the effects of physical activity or exercise. For cross‐sectional studies, recorded data on physical activity and/or sedentary time (device‐based, questionnaire) or cardiorespiratory fitness (oxygen consumption or surrogate measurement).
Biofluid	Blood (serum or plasma), saliva, urine
Exclusion criteria	
Language	Other than English
Document type	Other than original article (e.g., review or communication)
Exposure	Diet counseling, behavioral therapy, or other non‐exercise interventions.
Disease	Any endocrinological, metabolic or cardiovascular disease that could affect physical activity or body composition and thereby confound the associations or effects studied. Monogenetic causes of obesity and syndromic obesity.
Type of study	Genomics, transcriptomics, proteomics, and microbiomics.

### 2.3 Inclusion and Exclusion Criteria

Both cross‐sectional and longitudinal observational studies were considered eligible if conducted among children or adolescents aged 18 years or less and focused on either PA or sedentary time or CRF (Table 1). Intervention studies were eligible if conducted among individuals aged 18 years or less and exclusively focused on PA or sedentary time. Interventions with diet or other modes of therapy were excluded. The analytical focus was on metabolomics methods with either the highest compound coverage or the highest specificity (e.g., LC‐MS and NMR). Conventional methods with low specificities, such as colorimetry and immunoassays, and other omics approaches, such as genomics, transcriptomics, and proteomics, were excluded.

### 2.4 Data Collection and Synthesis

Metabolites that were statistically significantly altered in intervention studies or showed statistically significant associations with PA or CRF were included into a database along with their quantitative information (fold changes or standardized regression coefficients and their *p*‐values) and were annotated with the Kyoto Encyclopedia of Genes and Genomes (KEGG) and Human Metabolite Database (HMDB) identifiers, where applicable. Correspondence with metabolic functions and pathways from the Small Molecule Pathway Database (SMPDB) (Frolkis et al. 2009) was carried out using the enrichment analysis tool in the MetaboAnalyst 5.0 platform for the identified metabolites. Metabolic networks and pathway mappings were then visualized using the Metscape add‐on (version 3.1) from Cytoscape (version 3.9).

## 3 Results

### 3.1 Literature Search

**FIGURE 1:**
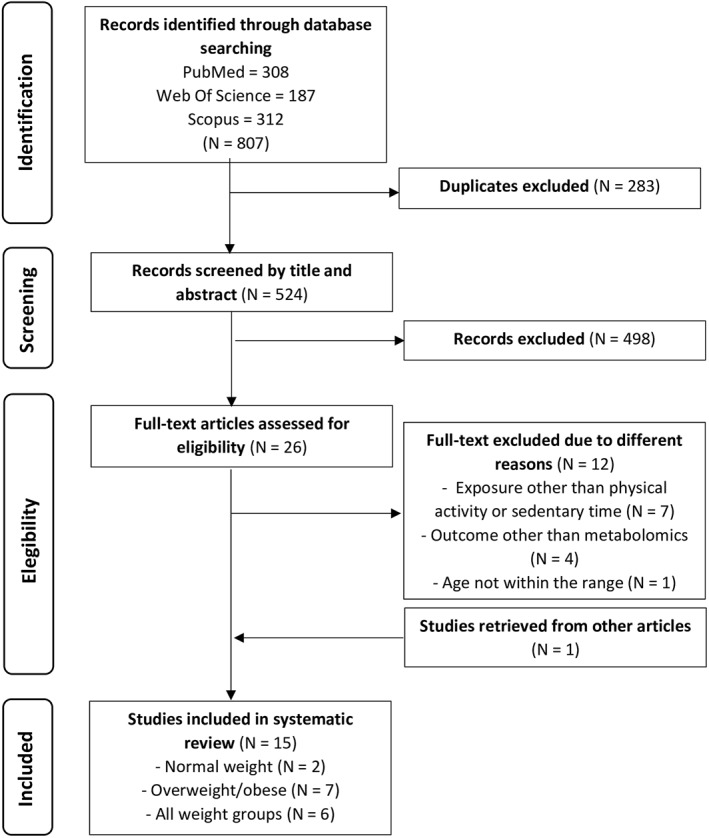
Flow chart of the systematic review.

The systematic search resulted in 14 articles considered eligible for the literature review (Zheng et al. 2014; Bell et al. 2018; Jones et al. 2019; Duft et al. 2022; Haapala, Leppänen, et al. 2022; Jones et al. 2021; Short et al. 2019; Zhou et al. 2021; Gumus et al. 2021; Rasooli et al. 2021; Duft et al. 2020; Meucci et al. 2017; Stergioulas and Filippou 2006; Wang et al. 2023). In addition, one article was further retrieved from secondary sources (Baghersalimi et al. 2019). The study selection followed the PRISMA statement presented in Figure 1. Of the 15 articles, 12 scored 70% or higher and were considered of adequate quality indicating a low risk of bias. All articles deemed eligible were included in the literature review.

### 3.2 Characteristics of the Studies

**FIGURE 2:**
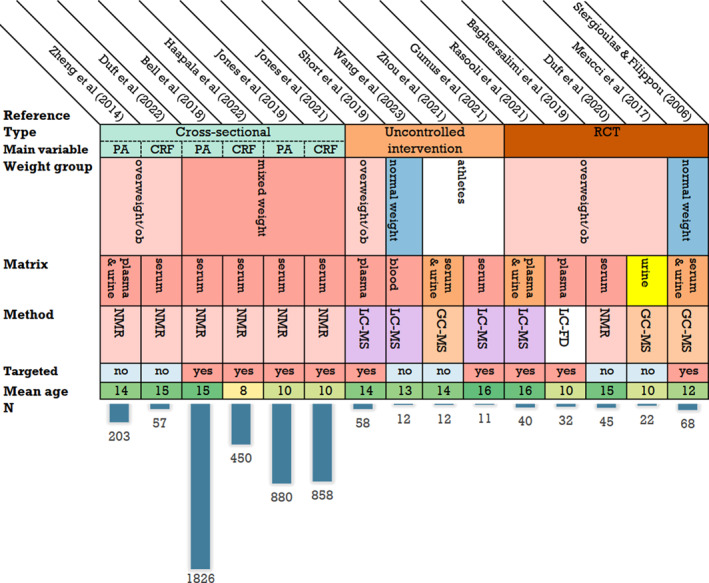
Summary of the studies included in this systematic review. CRF, Cardiorespiratory fitness; FD, Fluorescence detection; GC, Gas chromatography; LC, Liquid chromatography; MS, Mass spectrometry; NMR, Nuclear magnetic resonance; PA, Physical activity; RCT, Randomized controlled trial.

The characteristics of the studies included in the systematic review are summarized in Figure 2 and Table 2. Of the 15 studies, 5 were randomized controlled trials with PA as treatment (Rasooli et al. 2021; Duft et al. 2020; Meucci et al. 2017; Stergioulas and Filippou 2006; Baghersalimi et al. 2019) and 4 were uncontrolled intervention studies (Short et al. 2019; Zhou et al. 2021; Gumus et al. 2021; Wang et al. 2023). The number of participants in these 9 intervention studies ranged from 11 to 68. Of the 15 studies, 6 were cross‐sectional studies (Zheng et al. 2014; Bell et al. 2018; Jones et al. 2019; Duft et al. 2022; Haapala, Leppänen, et al. 2022; Jones et al. 2021): 3 focusing on PA (Zheng et al. 2014; Bell et al. 2018; Jones et al. 2019), and 3 on CRF (Duft et al. 2022; Haapala, Leppänen, et al. 2022; Jones et al. 2021). The number of participants in the cross‐sectional studies ranged from 57 to 1826. In all 15 studies, the age of the participants ranged from 6 to 18 years, with most of them having entered puberty. Seven studies were conducted among children or adolescents living with overweight or obesity (Zheng et al. 2014; Duft et al. 2022; Short et al. 2019; Rasooli et al. 2021; Duft et al. 2020; Meucci et al. 2017; Baghersalimi et al. 2019), two studies among those with normal weight (Stergioulas and Filippou 2006; Wang et al. 2023), 4 studies included all weight groups (Bell et al. 2018; Jones et al. 2019; Haapala, Leppänen, et al. 2022; Jones et al. 2021), and 2 studies were executed in athletes (Zhou et al. 2021; Gumus et al. 2021). Urine and blood (serum and plasma) were the 2 sample matrices represented, with 10 studies using blood (Bell et al. 2018; Jones et al. 2019; Duft et al. 2022; Haapala, Leppänen, et al. 2022; Jones et al. 2021; Short et al. 2019; Gumus et al. 2021; Duft et al. 2020; Wang et al. 2023; Baghersalimi et al. 2019), 1 study using urine (Meucci et al. 2017), and 4 studies using both (Zheng et al. 2014; Zhou et al. 2021; Rasooli et al. 2021; Stergioulas and Filippou 2006). NMR was used as the measurement method in 7 studies (Zheng et al. 2014; Bell et al. 2018; Jones et al. 2019; Duft et al. 2022; Haapala, Leppänen, et al. 2022; Jones et al. 2021; Duft et al. 2020), LC‐MS in 4 studies (Short et al. 2019; Gumus et al. 2021; Rasooli et al. 2021; Wang et al. 2023), and GC‐MS in 3 studies (Zhou et al. 2021; Meucci et al. 2017; Stergioulas and Filippou 2006). In addition, 1 study used LC with fluorescence detection (Baghersalimi et al. 2019). Of the studies, 5 applied an untargeted metabolomics approach, with 3 of these performed using NMR (Zheng et al. 2014; Duft et al. 2020, 2022) and 2 using GC‐MS (Zhou et al. 2021; Meucci et al. 2017).

**TABLE 2:** Characteristics of the studies included in the systematic literature review.

Reference	Study population, *n* of participants	Pubertal stage	Measurement technique	Sample matrix	Study type	Physical activity exposure	Main findings
Zheng et al. 2014	Denmark Ages 12–15 years Overweight *N* = 203	TS1–5	NMR Untargeted metabolites	Plasma, urine	Cross‐sectional	PA assessed by questionnaire and steps using pedometer.	No observed associations between PA and plasma or urine metabolites.
Bell et al. 2018	UK Mean age 15.4 ± 0.2 years Mixed weight, mean BMI 21.4 (SD 3.5) kg/m^2^ *N* = 1826	Not determined	NMR Targeted large‐scale analysis	Serum	Cross‐sectional	PA measured using a waist‐worn uniaxial ActiGraph, and expressed as mean minutes spent in total, SED and MVPA per day.	PA was negatively correlated with VLDL and chylomicron parameters and positively correlated with HDL parameters. Increased sedentary time was associated with decreased phenylalanine and tyrosine and increased alanine.
Jones et al. 2019	Norway Mean age 10 ± 0.3 years Mixed weight, mean BMI 18.1 (SD 3.0) kg/m^2^ *N* = 880	TS1–2 (88%) > TS2 (12%)	NMR Lipoprotein subclasses	Serum	Cross‐sectional	PA measured using a waist‐worn triaxial ActiGraph, and expressed as mean daily minutes spent in SED, Light PA, and MVPA.	MVPA was negatively correlated with VLDL and chylomicron particles and cholesterol independent of adiposity (waist circumference).
Duft et al. 2022	Brazil Mean age 14.5 years Overweight *N* = 57	TS4–5	NMR Untargeted metabolites	Serum	Cross‐sectional	VO2 peak used as a measure of CRF by treadmill test.	Serum glutamate, tyrosine, and valerate correlated negatively with CRF.
Haapala et al. 2022	Finland Mean age 7.6 ± 0.4 years Mixed weight *N* = 450	< TS2	NMR Targeted large‐scale analysis	Serum	Cross‐sectional	Maximal power output (Wmax) as a measure of CRF by a maximal exercise test + HR.	Serum glutamine and phenylalanine correlated positively with CRF. Also, HDL particles and cholesterol correlated positively with CRF.
Jones et al. 2021	Norway Mean age 10 ± 0.3 years Mixed weight *N* = 858	TS1–2 (93.4%) > TS2 (6.6%)	NMR Lipoprotein subclasses	Serum	Cross‐sectional	CRF measured using an intermittent shuttle run test, recording the total distance run by each child.	CRF was negatively correlated with VLDL and chylomicron particles and cholesterol independent of adiposity (waist circumference).
Short et al. 2019	USA Ages 11–17 years Normal and obese *N* = 94	≥ TS2	LC‐MS Amino acids	Plasma	Uncontrolled intervention	Intervention. Overweight children underwent a 48‐week exercise program subdivided into 3 phases. Each phase tested how different incentive structures would affect exercise frequency and/or duration.	No changes in amino acids observed during the intervention.
Zhou et al. 2021	China Mean age 13.7 ± 1.2 years Athletes, girls *N* = 12	Not determined	GC‐MS, w/derivatization Untargeted metabolites	Serum, urine	Uncontrolled intervention	Intervention. Study included 2 weeks integrity training cycle with 2 prime contents, aerobic endurance and the large‐load exercise. VO_2max_ on a treadmill using bruce + one‐repetition maximal.	Several metabolic changes. Serum amino acids, glycerol and fatty acids increased. Tricarboxylic acid cycle metabolites decreased in serum and urine.
Gumus et al. 2021	USA Ages 14–18 years Athletes *N* = 11	Mature adolescents	LC‐MS Amino acids	Plasma	Uncontrolled intervention	Intervention + HR + VO_2_. Maximal exercise test, a 1‐min incremental workload increased until volitional fatigue.	Increased in several glycolysis and fatty acid oxidation metabolites. Plasma levels of valine decreased and BCAA catabolism products increased in response to exercise.
Wang et al. 2023	China Mean age 12.9 ± 0.8 years Normal weight, boys *N* = 12	Not determined	LC‐MS Targeted lipidomics	Serum	Uncontrolled intervention	Intervention. Sprint interval training consisted of repeated Wingate tests on an ergometer, 3 days per week for 6 consecutive weeks.	Changes in 296 lipids, of which 33 changed significantly.
Duft et al. 2020	Brazil Aged 13–17 years Obese/overweight *N* = 45	TS4–5	NMR Untargeted metabolites	Serum	RCT	Intervention. Training consisted of resistance training and aerobic training in the same session, with a total of 60 min, three times a week, for 12 weeks.	Serum 2‐oxoisocaproate, 3‐hydroxyisobutyrate, glucose and pyruvate decreased while glutamine increased in the training group.
Meucci et al. 2017	USA Mean age 9.8 years Overweight *N* = 22	Not determined	GC‐MS, w/derivatization Untargeted metabolites	Urine	RCT	Intervention. Training group had 4 or 8 weeks of supervised play‐based activity, 6 h a day and 5 days per week. Control group spent a typical summer break.	In the 8‐week group, changes in several urine metabolites related to glycolysis, amino acid metabolism and purine degradation.
Stergioulas et al. 2006	Greece Mean age 12.1 years Normal weight, boys *N* = 68	Not determined	GC‐MS w/derivatization Lipids and arachidonic acid metabolites	Serum, urine	RCT	Intervention. Physical work capacity estimated based on measured height, weight and aerobic capacity. The training group performed 60 min of aerobic exercise 4 times per week for 8 weeks.	Urinary metabolites of prostacyclin and thromboxane decreased, and serum HDL increased in response to training.
Rasooli et al. 2021	Iran Ages 14–17 years Obese/overweight, boys *N* = 40	Not determined	LC‐MS Amino acids, glycine adducts	Plasma, urine	RCT	Intervention. 3 training sessions per week for 8 weeks. The training program consisted of 10 min of warm‐up, dedicated exercise (20–40 min) and 10 min of stretching to cooldown.	Plasma levels of glucose, valine, mannose, lysine and total BCAAs decreased and asparagine, glycine, serine increased in the training group. Urine levels of 2‐methylbutyrylglycine increased.
Baghersalimi et al. 2019	Iran Ages 9–11 years Obese/overweight, girls *N* = 32	TS2–3	LC‐FD w/derivatization Amino acids	Plasma	RCT	Intervention. Obese individuals divided into control, continuous walking or interval walking groups with 3 training sessions per week for 8 weeks. Control group was instructed to avoid exercise.	Plasma level of lysine increased in the control group

Abbreviations: BCAA, Branched‐chain amino acids; BMI, Body mass index; CRF, Cardiorespiratory fitness; FD, Fluorescence detection; FID, Flame ionization detection; GC, Gas chromatography; HDL, high density lipoprotein; HR, Heart rate; LC, Liquid chromatography; MS, Mass spectrometry; MVPA, Moderate‐to‐vigorous PA; NMR, Nuclear magnetic resonance; PA, Physical activity; RCT, Randomized controlled trial; SED, Sedentary time; TS, Tanner stage; VLDL, very‐low‐density lipoprotein; VO_2_, Oxygen uptake.

### 3.3 Effects of Randomized Controlled Physical Activity Interventions on Metabolites

Nine studies investigated the effects of different PA interventions on blood or urine metabolites with a total of 300 participants (Table 2). The structures of the interventions are detailed further in Supporting Information S1: Table S5. Rasooli et al. (2021) investigated the effects of an 8‐week circuit resistance training on plasma metabolites and urinary glycine‐conjugated adducts using LC‐MS in 40 adolescent boys living with obesity, divided into a training group and a control group. Plasma glucose, valine, mannose, lysine, and total branched‐chain amino acids (BCAAs) decreased, while plasma asparagine, glycine, and serine increased in the training group compared to the control group. In addition, urinary 2‐methylbutyrylglycine and butyrylglycine increased in the training group compared to the control group, which the authors attributed to increased amino acid degradation. Baghersalimi et al. (2019) investigated the effects of an 8‐week walking program on plasma amino acids using LC with fluorescence detection in 32 girls aged 9–11 years living with obesity, divided into a continuous‐walking group, an interval‐walking group, and a control group. While most amino acids were unaltered in response to the walking interventions, lysine increased in the control group but not in the walking groups. In addition, global arginine bioavailability, which the authors defined as the sum of arginine, citrulline, and ornithine, decreased in the control and interval‐walking group but not in the continuous‐waking group. Duft et al. (2020) investigated the effects of a 12‐week combined resistance and aerobic training on serum metabolites using an untargeted NMR analysis in 37 adolescents living with overweight or obesity. 2‐oxoisocaproate, 3‐hydroxyisobutyrate, glucose, and pyruvate decreased, while glutamine increased in the training group compared to the control group. Stergioulas and Filippou (2006) investigated the effects of an 8‐week endurance training followed by a 4‐week detraining period on urinary lipids and arachidonic acid metabolites using GC‐MS in 68 adolescent boys living with normal weight. A major urinary metabolite of prostacyclin decreased in the intervention group but not in the control group. Meucci et al. (2017) studied the effects of a 4‐ or 8‐week supervised play‐based PA intervention on the urinary metabolomic signature using untargeted GC‐MS in 22 children and adolescents aged 8–12 years living with overweight. I 8‐week intervention changed several urine metabolites related to glycolysis, amino acid metabolism, and purine degradation compared to the control group.

### 3.4 Effects of Uncontrolled Physical Activity Interventions on Metabolites

Wang et al. (2023) studied the effects of a 6‐week sprint interval training with no control group on circulatory lipid profiles in 12 untrained adolescent boys living with normal weight. Serum ceramides and several long‐chain diglycerides increased and free long‐chain fatty acids decreased during the intervention. Short et al. (2019) studied the effects of a 16‐week training program consisting of 20 min of any type of MVPA per week at a fitness center with no intervention group on plasma amino acids and derivatives in 58 adolescents living with overweight. and found no changes in amino acids during the intervention. Zhou et al. (2021) studied the metabolic effects of 2‐week strength‐endurance training among 12 female adolescent athletes with no control group using an untargeted GC‐MS analysis. Several serum amino acids, glycerol, and fatty acids increased, while serum and urine metabolites of the citric acid cycle decreased in response to exercise. Gumus et al. (2021) investigated the effects of a single bout of maximal aerobic exercise on targeted plasma metabolites using LC‐MS in 11 adolescent athletes. Plasma glycerol, ketones, *β*‐hydroxybutyrate, acylcarnitines, pyruvate, lactate, alanine, glutamate, and BCAA catabolism products increased and plasma valine decreased in response to the acute exercise.

### 3.5 Associations Between Measures of Physical Activity and Metabolites

Three cross‐sectional studies investigated the associations between PA and blood metabolites with a total of 2909 participants (Table 2). Zheng et al. (2014) found no associations of daily steps taken with plasma or urine metabolites analyzed by untargeted NMR in 203 adolescents living with overweight. Bell et al. (2018) studied the associations of device‐measured total PA with serum lipids and other metabolites, including amino acids and fatty acids, using targeted NMR in 1826 adolescents with mixed weight, in which 4.2% were living with obesity. Higher device‐measured total PA was associated with a more favorable lipid profile, entailing higher HDL cholesterol and particle size, lower very‐low‐density lipoprotein (VLDL) cholesterol and particle count, and lower triglycerides. Total PA was also associated with lower alanine and pyruvate and higher aromatic amino acids and citrate. A higher MVPA was additionally associated with a lower 3‐hydroxybutyrate, and higher sedentary time was associated with lower tyrosine and citrate as well as with higher alanine, histidine, and creatinine. Jones et al. (2019) studied the associations of device‐measured MVPA with serum lipids using NMR in 880 adolescents across all weight groups. Higher device‐measured MVPA was associated with a more favorable lipid profile entailing lower VLDL and chylomicron particles, lower cholesterol and triglycerides in these lipoproteins, and lower total triglycerides independent of waist circumference. Furthermore, higher sedentary time was associated with larger VLDL particles and chylomicron cholesterol content and smaller average LDL particle size. However, these associations were largely explained by waist circumference.

### 3.6 Associations Between Cardiorespiratory Fitness and Metabolites

Three studies investigated the associations between CRF and blood metabolites with a total of 1365 participants (Table 2). Duft et al. (2022) studied 33 adolescents living with overweight and found that CRF, assessed by VO_2_ peak divided by body mass, was negatively associated with serum glutamate, tyrosine, and valerate analyzed with untargeted NMR. Haapala, Leppänen, et al. (2022) studied 450 children from a general population, in which 13% were living with overweight, and found that CRF, assessed by a maximal workload divided by lean mass, was positively associated with serum glutamine and phenylalanine, analyzed by the same targeted NMR as (Bell et al. 2018). In addition, CRF was positively associated with medium‐sized HDL particles and HDL cholesterol. Jones et al. (2021) investigated the associations of CRF, assessed by an intermittent shuttle run test, with serum lipoprotein subclasses using NMR in 858 adolescents from the same cohort as in the other study (Jones et al. 2019). They observed that CRF was negatively associated with VLDL and chylomicron particles, cholesterol in these lipoproteins, and total triglycerides independent of waist circumference (Jones et al. 2021).

### 3.7 Enrichment and Pathway Analysis

**FIGURE 3:**
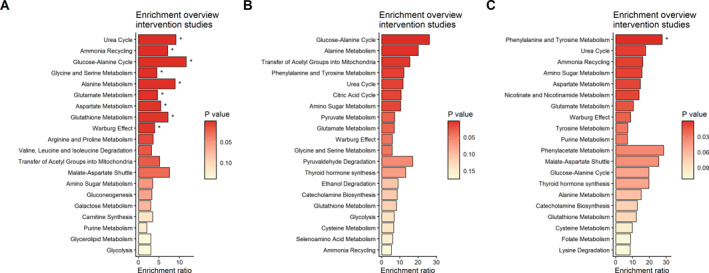
Pathway analysis results upon inclusion of: (A) metabolites with significant changes in the intervention studies, (B) metabolites with significant associations with physical activity, and (C) metabolites with significant associations with cardiorespiratory fitness. Metabolites were compared to SMPDB pathways using MetaboAnalyst 5, Enrichment Analysis. * Indicates a statistically significant enrichment of the pathway (*p* < 0.05 after false discovery rate correction).

Following the enrichment analysis, it was observed that the circulating metabolites mainly comprised of amino acids and their metabolites, and particularly glycogenic amino acids (Figure 3). The affected pathways in the PA intervention studies (*p* < 0.05 after false discovery rate correction) were the urea cycle and ammonia recycling, followed by the glucose‐alanine cycle, the metabolism of amino acids glycine, serine, alanine, glutamate, and aspartate, glutathione metabolism, and aerobic glycolysis, also known as the Warburg effect. When looking at the metabolites associated with CRF, the phenylalanine‐tyrosine metabolic pathway was enriched. The glucose‐alanine cycle and alanine metabolism were the most enriched pathways associated with PA, although the results were not statistically significant after the false discovery rate correction. Metabolic networks and pathway mappings are visualized in Figure S1.

## 4 Discussion

**FIGURE 4:**
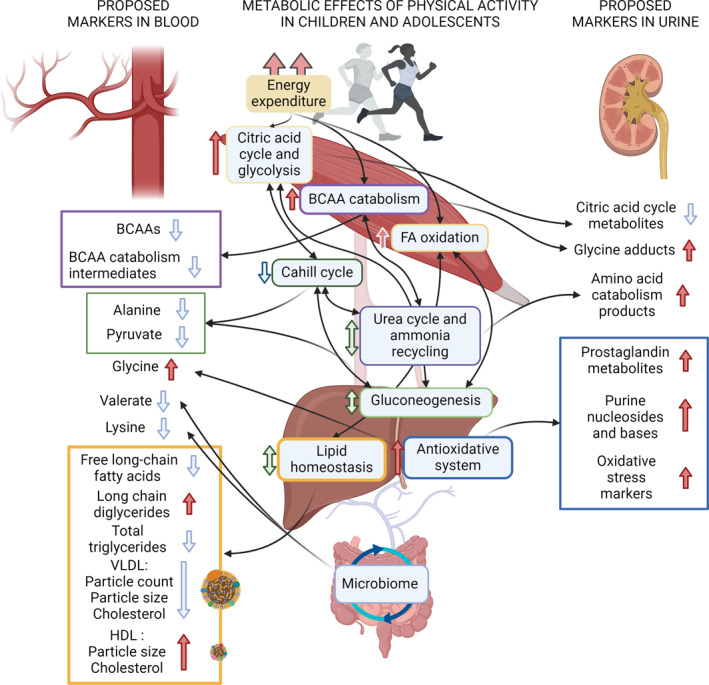
Summary of the main findings of this systematic review. BCAA, branched‐chain amino acids; FA, fatty acid; HDL, high density lipoprotein; VLDL, very‐low‐density lipoprotein. Created with Biorender.com.

The aim of this work was to systematically review the literature on the effects of PA interventions on the metabolome and the associations of PA, sedentary time, and CRF with the metabolome of children and adolescents as well as to address the role of weight status in this context. We found 15 studies on this topic, half of which were conducted in individuals living with overweight. Notably, most of these studies used metabolomics methods targeting lipid subgroups or amino acids. The absence of metabolic fingerprinting studies using LC‐MS indicates a niche for these studies in the fields of pediatrics, exercise science, and public health. Nevertheless, using manual curation and pathway analysis, we identified metabolites and metabolic pathways that could serve as potential biomarkers of the beneficial effects of PA on cardiometabolic health, among these lipid metabolism, BCAA catabolism, ammonia metabolism, and the glucose‐alanine cycle (Figure 4). The interconnectivity between amino acid metabolism, ammonia degradation, and the central energy metabolism of skeletal muscle was elucidated by metabolic networks (Figure S1). The directions of changes in metabolites during PA interventions or metabolites associated with PA were largely consistent across children and adolescents with normal body weight and those living with overweight.

High‐intensity PA caused acute depletion of serum long‐chain acyl‐carnitines and an increase in fatty acid oxidation products (Gumus et al. 2021), and likewise, an increase in free long‐chain fatty acids (Zhou et al. 2021), corroborating previous research (Hargreaves and Spriet 2020) (Figure 4). Higher PA and CRF were also consistently associated with a more favorable blood lipid profile entailing lower triglycerides (Bell et al. 2018; Jones et al. 2019; Stergioulas and Filippou 2006), larger and more lipid‐rich HDL particles, and higher Apo‐A1 (Bell et al. 2018; Jones et al. 2019; Duft et al. 2022; Haapala, Leppänen, et al. 2022). Besides HDL, the VLDL metabolism appeared to be PA‐dependent in studies (Bell et al. 2018; Jones et al. 2019; Duft et al. 2022), seemingly opposing changes seen in type 2 diabetes in pediatric populations (Tricò et al. 2018). Our results largely corroborate previously found associations of higher PA and better CRF with improved lipid subclass profiles among adults (Kujala et al. 2019, 2022, 2013) and adolescents (Lehtovirta et al. 2022). Higher PA associates with more favorable blood lipid profiles and can thereby induce beneficial effects on cardiometabolic risk at an early age. High‐quality experimental studies should elucidate these causal links further.

PA decreased serum concentrations of total BCAAs, valine (Gumus et al. 2021; Rasooli et al. 2021) and 2‐oxoisocaproate and 3‐hydroxyisobutyrate (Duft et al. 2020), respective catabolic intermediates of leucine and valine (Yudkoff et al. 2005; Jang et al. 2016). In urine, glycine‐conjugated adducts (Rasooli et al. 2021) and 2‐hydroxy‐3‐methylbutyric acid (Meucci et al. 2017), derived from BCAA catabolism (Liebich and Först 1984), tended to increase. BCAAs are important substrates for skeletal muscle tissue (Zhou et al. 2021), providing substrates for central energy production, and their catabolism may also regulate a shift from glycolysis to beta‐oxidation as an energy source (Kainulainen et al. 2013). However, consistently elevated circulating levels of BCAAs have been observed in obesity and type 2 diabetes (Lynch and Adams 2014). The serum level of the catabolic intermediate of valine, 3‐hydroxyisobutyrate, is considered a novel predictor of type 2 diabetes (Sacks et al. 2018). PA can potentially alleviate the overweight‐induced changes in BCAA metabolism in early life, which warrants further studies.

The urea cycle and ammonia recycling pathways were affected by PA interventions (Figure 3) and centrally associated with the metabolism of amino acids (Figure S1). Excess ammonia, which is not excreted as urea, may alternatively be recycled in series of transamination and deamination reactions between amidic and dicarboxylic amino acids (Walls et al. 2015; Batool et al. 2016). Arginine, which is catabolized to urea and ornithine, has been found to have ergogenic and cardioprotective effects through the supply of nitric oxide, which improves vascular endothelial function (Hargreaves and Spriet 2020; Gornik and Creager 2004). Higher PA and CRF also tended to associate with higher circulating glutamine and asparagine as well as with lower glutamate and aspartate (Haapala, Leppänen, et al. 2022; Zhou et al. 2021; Rasooli et al. 2021; Duft et al. 2020). Decreased circulating glutamine levels have previously been associated with childhood obesity (De Spiegeleer et al. 2021), and both glutamine and asparagine have been suggested as substrates against fatigue (Marquezi et al. 2003; Coqueiro et al. 2019). PA may possibly induce beneficial changes in ammonia metabolism in children and adolescents, but this awaits further confirmation.

PA notably affected the metabolic pathways involving alanine, glucose, and glutamate (Figure 3). A single bout of vigorous PA increased serum alanine and glutamate (Zhou et al. 2021; Gumus et al. 2021), while in the cross‐sectional studies, higher PA and CRF were associated with lower serum alanine, glutamate, and pyruvate (Bell et al. 2018; Duft et al. 2022). This may be due to adaptive mechanisms and increased uptake of these metabolites into the liver and skeletal muscle (Sarabhai and Roden 2019). Higher concentrations of this trio have been associated with insulin resistance in childhood (De Spiegeleer et al. 2021). These metabolites participate in the glucose‐alanine cycle, which acts as a way to degrade muscle proteins to provide substrates for gluconeogenesis (PubChem Pathway Summary for PathwayBlob SMP0087221 2022). PA interventions also lowered fasting plasma glucose (Duft et al. 2022; Rasooli et al. 2021), although this observation itself is not novel (Sampath Kumar et al. 2019). PA increases energy demand and may consequently upregulate central energy production pathways (Gibala et al. 1998). While a single bout of exercise in adolescents increased blood pyruvate circulation (Gumus et al. 2021), long‐term studies showed an inverse relationship between PA and pyruvate (Bell et al. 2018; Duft et al. 2020) and direct relationship with PA and citrate (Bell et al. 2018). These results generally contradict the changes observed in obesity and metabolic dysfunctions (Butte et al. 2015; De Spiegeleer et al. 2021). Taken together, the findings suggest additional mechanisms by which PA could influence muscle and liver metabolism at an early age (Pedersen and Febbraio 2012) (Figure 4).

Metabolic pathways involving glycine and glutathione were found to be altered in interventions (Figure 3). Both circulating oxidized glutathione and glutathione breakdown products (Mullins et al. 2020), increased in response to high‐intensity exercise (Zhou et al. 2021). Urinary release of biomarkers of oxidation also tended to increase in response to exercise (Zhou et al. 2021; Meucci et al. 2017). This may be due to PA having conditioning effects on glutathione‐dependent antioxidant defenses (Sen 1999), which could potentially counteract the oxidative states seen in childhood obesity and diabetes (Pastore et al. 2012). In addition, the PA‐induced increase in serum glycine (Zhou et al. 2021; Rasooli et al. 2021) could support the synthesis and degradation of glutathione. Indeed, lower circulating levels of glycine have been observed in children with obesity and impending insulin resistance (De Spiegeleer et al. 2021; Rasooli et al. 2021), making the increase of this biomarker by PA another interesting finding.

The cross‐sectional associations of PA and CRF with the circulating aromatic amino acids (Figure 3), have been examined in several studies among children living with obesity (De Spiegeleer et al. 2021). The results of these studies have been somewhat contradictory for tyrosine, which was negatively associated with CRF (Duft et al. 2022) and positively associated with total PA (Bell et al. 2018). In other cross‐sectional studies among children and adolescents, CRF (Haapala, Leppänen, et al. 2022) and PA (Bell et al. 2018) were directly related to phenylalanine. However, no changes in aromatic amino acids were seen in any of the intervention studies. Aromatic amino acids, have been reported to be higher in children with obesity compared to peers with normal body weight (Butte et al. 2015). In addition, higher circulating aromatic amino acid levels may predict increases in BMI and fat mass in adolescents (Rodríguez‐Carmona et al. 2022). As exercise has been shown to mitigate oxidative stress caused by high levels of phenylalanine in rats (Mazzola et al. 2011), it is possible that more active individuals are tolerant of higher phenylalanine levels and its associated oxidative stress. Further research should confirm if PA has predictable effects on aromatic amino acids in pediatric populations, and whether body composition or biological maturation may confound these results.

Only two studies were found to investigate alterations in metabolites associated with sedentary time. Higher sedentary time associated with lower tyrosine and citrate as well as with higher alanine, histidine, and creatinine in mixed‐weight adolescents (Bell et al. 2018), generally showing opposite associations as for PA. Yet, sedentary time was not associated with any lipid measures independent of adiposity (Jones et al. 2019). Together with lower PA, higher sedentary time has been associated with increased cardiometabolic risk since childhood, although the modulatory effects of physical inactivity on cardiometabolic health have not been fully elucidated (Lavie et al. 2019). Therefore, more studies on the metabolic effects of sedentary time in children and adolescents are warranted.

This review covered children and adolescents with varying body weight, while half of the studies were conducted in participants living with overweight. Thus, in the cross‐sectional studies, only associations that were controlled for adiposity measures, such as fat mass or waist circumference, were of interest for this review. Using this approach, the associations of PA with several circulating metabolites, such as triglycerides and VLDL‐markers, were found remarkably consistent across studies. Unfortunately, a subgroup analysis for normal‐ and overweight populations was not feasible due to the small number of studies. The two studies conducted in adolescent athletes were somewhat outliers in this review. However, they give insight into how different intensities of PA interventions and different baseline characteristics of the participants can affect metabolomics results in adolescents.

Urine, a commonly used sample matrix in metabolomics, was used in four PA‐intervention studies on adolescents. While the analysis methods and participants varied across these studies, the shifts seen in urinary metabolites seemingly corroborated the PA‐associated increase in BCAA catabolism, upregulation of the citric acid cycle and increase in oxidative stress (Figure 4). Further studies on urine metabolites with larger numbers of participants and greater compound coverage are needed to confirm these findings. Besides urine, saliva could also offer a great alternative for researchers and clinicians to easily, non‐invasively, and repeatedly assess changes in biomarkers and even predict the development of cardiometabolic diseases since childhood (Shah 2018; Wijnant et al. 2020). Surprisingly, none of the studies included in the current review used salivary metabolomics, highlighting the need for this in future studies.

The main strength of this systematic review was the use of a comprehensive search strategy, including subjective and device‐based measures of PA and sedentary time, and the additional strength of using measures of CRF. The inclusion of several study designs and articles published since inception ensured broad coverage. A limitation of the review is the relatively small number of studies based on different methodologies that were available. In addition, since the intervention studies had relatively small sample sizes (< 95 participants) and not all included a control group, the results should be confirmed in high‐quality trials with larger sample sizes. There were only few studies applying untargeted metabolomics, with a notable absence of untargeted LC‐MS analyses, which might limit drawing conclusions about metabolic alterations associated with PA and CRF. In addition, the different ways of assessing PA might partly explain the heterogeneity in the results of studies. Furthermore, several studies included children and adolescents at different stages of pubertal development and not all of them took puberty into account in the statistical analyses although it may affect the levels of PA and sedentary time, exercise responses, and metabolic profiles. Finally, only two studies were conducted in children, referring to the participants under 10 years in accordance with the WHO (World Health Organization 2023), limiting the generalization of the findings below that age. To gain knowledge on pediatric populations in various developmental phases, further studies are warranted.

Depending on the type and intensity, PA induced a multitude of acute and long‐term metabolic changes related to muscle energy balance, amino acid turnover, and antioxidant systems. Moreover, across children and adolescents of different weight groups, increased PA seemed to be associated with changes in circulating BCAAs, lipids, and citric cycle metabolites, which generally seemed to oppose the changes seen in obesity and insulin resistance at an early age. Further studies could corroborate whether the observed effects and relationships are causal in nature. In addition, baseline PA level, exercise modalities, body composition (e.g., body fat and lean mass), and biological maturation should be considered in future studies. Overall, the field of metabolomics in exercise medicine gravely lacks research on pediatric populations. Novel, accessible sample matrices, such as saliva, would make it easier to collect samples and increase sample sizes for metabolomics studies. Finally, global metabolomic fingerprinting methods with high sensitivity and coverage, such as high‐resolution MS, represent a special niche in the fields of pediatrics, exercise science, and public health.

## Funding

This research was funded by the Research Council of Finland (MHL: grant nr 350820) and supported by the Swedish Cultural Foundation in Finland (HV) and Folkhälsan Research Foundation (HV).

## Conflicts of Interest

The authors declare no conflicts of interest.

## Supporting information


Supporting Information S1

## Data Availability

The protocol was registered in the International Prospective Register of Systematic Reviews (PROSPERO, id 366821) prior to searches and analyses being completed. All relevant data for this article is included in the main text or as supplementary files.
