# Supplementary material for: Physical Activity and Metabolic Alterations in Children and Adolescents Across Different Weight Groups: A Systematic Review
Source: Eur J Sport Sci. 2026 Jun 20;26(7):e70206. doi: 10.1002/ejsc.70206 (PMC13283064; doi:10.1002/ejsc.70206)
Supplement: Supplementary file 1 — Supporting Information S1 [file EJSC-26-e70206-s001.docx]

**Physical activity and metabolic alterations in children and adolescents across different weight groups: A systematic review
2024**

**Table S1.** Search strategy used for this systematic review in three different databases.

| **Database** | **Search strategy** |
| --- | --- |
| **Pubmed** | ((children OR childhood OR paediatric OR adolescents OR under-18 OR teenage OR youngster OR ”boy” OR ”girl”)  AND (physical activity OR exercise OR sedentary time OR sedentariness OR “cardiorespiratory fitness” OR "aerobic capacity” OR "aerobic fitness” OR physical fitness OR "oxygen consumption” OR “oxygen uptake” OR "VO_2_max” OR "VO_2_peak” OR fitness)  AND ((blood OR serum OR plasma OR urine OR saliva) AND (metabolomics OR metabolites OR metabolome OR “metabolic profil*” OR “metabolic fingerprint*” OR “lipidom*”))  AND (weight OR overweight OR “body mass index” OR BMI OR “fat mass” OR obesity OR adiposity)  AND english[Language]  AND ("1900"[Date - Publication]: "2023/12/31"[Date - Publication])  NOT ("review"[Publication Type] OR "systematic review"[Publication Type] OR "systematic review"[Title] OR "meta-analysis"[Publication Type])) |
| **Web of Science** | (ALL=(children OR childhood OR paediatric OR paediatric OR adolescents OR adolescence OR teenage OR youngsters OR under-18 OR ”boy” OR ”girl”)  AND ALL=(”physical activity” OR exercise OR (“sedentary time” OR “sedentary behavior”) OR sedentariness OR “cardiorespiratory fitness” OR "aerobic capacity” OR "aerobic fitness” OR “physical fitness” OR "oxygen consumption” OR “oxygen uptake” OR VO2max OR VO2peak OR fitness)  AND ALL=((blood OR serum OR plasma OR urine OR saliva) AND (metabolomics OR metabolites OR metabolome OR “metabolic profil*” OR “metabolic fingerprint*” OR “lipidom*”))  AND ALL=(weight OR overweight OR “body mass index” OR BMI OR “fat mass” OR obesity OR adiposity)  AND LA=(English)  AND DOP=(1900-01-01/2023-12-31)  NOT (DT=(Review) OR TI=(”systematic review”))) |
| **Scopus** | ((TITLE-ABS-KEY(children OR childhood OR paediatric OR adolescents OR adolescence OR teenage OR youngster OR “under-18” OR ”boy” OR ”girl”)  AND TITLE-ABS-KEY(”physical activity” OR exercise OR “sedentary time” OR “sedentary behavior” OR sedentariness OR “cardiorespiratory fitness” OR "aerobic capacity” OR "aerobic fitness” OR “physical fitness” OR "oxygen consumption” OR “oxygen uptake” OR VO_2_max OR VO_2_peak OR fitness)  AND (TITLE-ABS-KEY(blood OR serum OR plasma OR urine OR saliva) AND TITLE-ABS-KEY(metabolomics OR metabolites OR metabolome OR “metabolic profile” OR “metabolic fingerprint” OR “lipidom*”))  AND TITLE-ABS-KEY(weight OR overweight OR “body mass index” OR BMI OR “fat mass” OR obesity OR adiposity)  AND LANGUAGE(English)  AND DOCTYPE(ar))  AND (EXCLUDE(PUBYEAR,2024))) |

|  | **Cross-sectional studies** | | | | | |
| --- | --- | --- | --- | --- | --- | --- |
|  | Bell *et al* | Duft *et al* | Haapala *et al* | Jones *et al* | Zheng *et al* | Jones *et al* |
| **1. Were the criteria for inclusion in the sample clearly defined?** | *Yes* | *Yes* | *Yes* | *Yes* | *Yes* | *Yes* |
| **2. Were the study subjects and the setting described in detail?** | *Yes* | *Yes* | *Yes* | *Yes* | *Yes* | *Yes* |
| **3. Was the exposure measured in a valid and reliable way?** | *Yes* | *Yes* | *No* | *Yes* | *No* | *No* |
| **4. Were objective, standard criteria used for measurement of the condition?** | *Yes* | *Yes* | *Yes* | *Yes* | *Yes* | *Yes* |
| **5. Were confounding factors identified?** | *Yes* | *No* | *Yes* | *Yes* | *Yes* | *Yes* |
| **6. Were strategies to deal with confounding factors stated?** | *Yes* | *No* | *Yes* | *Yes* | *Yes* | *Yes* |
| **7. Were the outcomes measured in a valid and reliable way?** | *Yes* | *Yes* | *Yes* | *Yes* | *Yes* | *Yes* |
| **8. Was appropriate statistical analysis used?** | *Yes* | *Yes* | *Yes* | *Yes* | *Yes* | *Yes* |
| **N of [yes] responses** | 8 | 6 | 7 | 8 | 7 | 7 |
| **N of questions** | 8 | 8 | 8 | 8 | 8 | 8 |
| **Score** | 1.00 | 0.75 | 0.88 | 1.00 | 0.88 | 0.88 |

**Table S2.** Quality and risk of bias assessment for the cross-sectional studies included in the systematic review. Scale used is the checklist for cross-sectional studies by the Joanna Briggs Institute. Quality score is calculated as n [YES] / (n[total] – n [NA]).

**Table S3.** Quality and risk of bias assessment for the randomized controlled trials (RCT) included in the systematic review. Scale used is the checklist for RCTs by the Joanna Briggs Institute. Quality score is calculated as n [YES] / (n[total] – n [NA]).

|  | **Randomized controlled trials** | | | | |
| --- | --- | --- | --- | --- | --- |
|  | Rasooli *et al* | Baghersalimi *et al* | Duft *et al* | Meucci *et al* | Stergioulas *et al* |
| **1. Was true randomization used for assignment of participants to treatment groups?** | *Yes* | *Yes* | *Yes* | *No* | *Yes* |
| **2. Was allocation to treatment groups concealed?** | *Unclear* | *Unclear* | *Unclear* | *Unclear* | *Unclear* |
| **3. Were treatment groups similar at the baseline?** | *Yes* | *Yes* | *Yes* | *Yes* | *Yes* |
| **4. Were participants blind to treatment assignment?** | *NA* | *NA* | *NA* | *NA* | *NA* |
| **5. Were those delivering treatment blind to treatment assignment?** | *NA* | *NA* | *NA* | *NA* | *NA* |
| **6. Were outcomes assessors blind to treatment assignment?** | *NA* | *NA* | *NA* | *NA* | *NA* |
| **7. Were treatment groups treated identically other than the intervention of interest?** | *Yes* | *Yes* | *Yes* | *Yes* | *Yes* |
| **8. Was follow up complete and if not, were differences between groups in terms of their follow up adequately described and analyzed?** | *No* | *No* | *No* | *Yes* | *No* |
| **9. Were participants analyzed in the groups to which they were randomized?** | *Yes* | *Yes* | *Yes* | *Yes* | *Yes* |
| **10. Were outcomes measured in the same way for treatment groups?** | *Yes* | *Yes* | *Yes* | *No* | *Yes* |
| **11. Were outcomes measured in a reliable way?** | *Yes* | *Yes* | *Yes* | *Yes* | *Yes* |
| **12. Was appropriate statistical analysis used?** | *Yes* | *Yes* | *Yes* | *Yes* | *Yes* |
| **13. Was the trial design appropriate, and any deviations from the standard RCT design (individual randomization, parallel groups) accounted for in the conduct and analysis of the trial?** | *Yes* | *Yes* | *Yes* | *Unclear* | *Yes* |
| **N of [yes] responses** | 7 | 7 | 7 | 5 | 7 |
| **N of questions** | 10 | 10 | 10 | 10 | 10 |
| **Score** | 0.70 | 0.70 | 0.70 | 0.50 | 0.70 |

**Table S4.** Quality and risk of bias assessment for the uncontrolled intervention studies included in the systematic review. Scale used is the checklist for quasi-experimental studies by the Joanna Briggs Institute. Quality score is calculated as n [YES] / (n[total] – n [NA]).

|  | **Intervention studies** | | | |
| --- | --- | --- | --- | --- |
|  | Short *et al* | Zhou *et al* | Gumus *et al* | Wang *et al* |
| **1. Is it clear in the study what is the ‘cause’ and what is the ‘effect’ (i.e. there is no confusion about which variable comes first)?** | *Yes* | *Yes* | *Yes* | *Yes* |
| **2. Were the participants included in any comparisons similar?** | *Yes* | *Yes* | *Yes* | *Yes* |
| **3. Were the participants included in any comparisons receiving similar treatment/care, other than the exposure or intervention of interest?** | *Unclear* | *NA* | *NA* | *NA* |
| **4. Was there a control group?** | *Yes* | *No* | *No* | *No* |
| **5. Were there multiple measurements of the outcome both pre and post the intervention/exposure?** | *No* | *No* | *No* | *No* |
| **6. Was follow up complete and if not, were differences between groups in terms of their follow up adequately described and analyzed?** | *No* | *Yes* | *Yes* | *Yes* |
| **7. Were the outcomes of participants included in any comparisons measured in the same way?** | *NA* | *NA* | *NA* | *NA* |
| **8. Were outcomes measured in a reliable way?** | *Yes* | *Yes* | *Yes* | *Yes* |
| **9. Was appropriate statistical analysis used?** | *Yes* | *Yes* | *Yes* | *Yes* |
| **N of [yes] responses** | 5 | 5 | 5 | 5 |
| **N of questions** | 8 | 7 | 7 | 7 |
| **Score** | 0.63 | 0.71 | 0.71 | 0.71 |

**Table S5.** Details of the physical activity programs in the intervention studies.

| **Reference** | **Age** | **Weight group** | **Physical activity type** | **Duration,**  **Weekly sessions** | **Intensity** |
| --- | --- | --- | --- | --- | --- |
| Short *et al* | 11-18 yrs | Overweight/  obese | Combined training, unsupervised | 16 weeks  2 x 34 min | MVPA, self-reported |
| Zhou *et al* | 12-14 yrs | Athletes | Aerobic and large-load resistance training | 2 weeks  11 x 80 min | 70 % VO_2max_, 70% 1RM |
| Gumus *et al* | 11-18 yrs | Athletes | Single 1-min incremental workload increased until volitional fatigue | Single time, average duration 12 min | Until fatigue |
| Rasooli *et al* | 14-17 yrs | Overweight | Circuit resistance training | 8 weeks  3 x 50 min | 70-80% 1RM |
| Baghersalimi *et al* | 11-18 yrs | Overweight | Continuous walking or interval walking | 8 weeks  3x 27 min | 60-75%, then 75-85 % HR_max_ |
| Duft *et al* | 11-18 yrs | Overweight | Combined training | 12 weeks  3 x 60 min | 50-85% VO_2Max_, 60-70% 1RM |
| Meucci *et al* | 10 yrs | Overweight | Supervised play-based activity | 8 weeks  5 x 360 min | MVPA, self-reported |
| Stergioulas *et al* | 10-14 yrs | Normal weight | Aerobic ergometer training | 8 weeks  4 x 60 min | 80% from physical work capacity |
| Wang *et al* | 12-14 yrs | Normal weight | Sprint interval training on ergometer | 6 weeks  3 x 40 min | Ergometer resistance 7.5% of body weight |

Abbreviations: MVPA = moderate-to-vigorous physical activity, VO_2max_ = peak oxygen uptake, 1RM = one-repetition maximal, HR = heart rate.


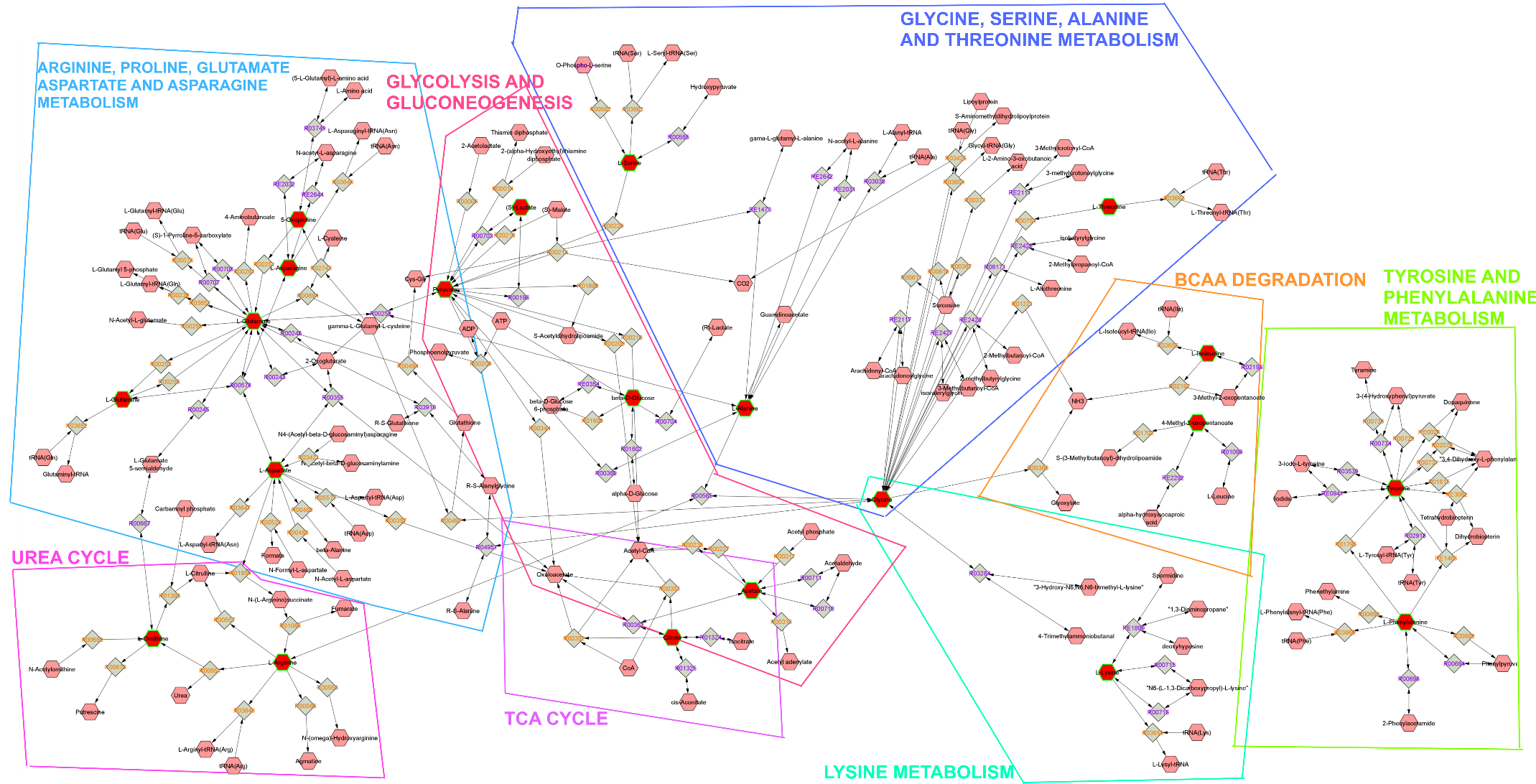


**Figure S1.** Metabolic networks as visualized with Metscape 3 with pathway mapping annotated post hoc. Hexagonal nodes signify compounds, diamond nodes reactions. Nodes in dark red with green outline were user-inputted, e.g., significantly altered in one or more studies. The figure shows the interdependence of central energy metabolism, glycolysis and TCA cycle (citric acid cycle), and the metabolism of several amino acids. Abbreviations: BBCA = branched-chain amino acids, TCA = tricarboxylic acid.
